# Abnormal cortical thickening and thinning in idiopathic normal-pressure hydrocephalus

**DOI:** 10.1038/s41598-020-78067-x

**Published:** 2020-12-03

**Authors:** Kyunghun Kang, Jaehwan Han, Sang-Woo Lee, Shin Young Jeong, Yong-Hyun Lim, Jong-Min Lee, Uicheul Yoon

**Affiliations:** 1 Department of Biomedical Engineering, Hanyang University, Seoul, South Korea; 2 Department of Neurology, School of Medicine, Kyungpook National University, Daegu, South Korea; 3 Department of Biomedical Engineering, Daegu Catholic University, Gyeongsan-si, South Korea; 4 Department of Nuclear Medicine, School of Medicine, Kyungpook National University, Daegu, South Korea; 5 Center of Self-Organizing Software-Platform, Kyungpook National University, Daegu, South Korea

**Keywords:** Neurodegenerative diseases, Diseases of the nervous system, Dementia

## Abstract

We investigated differences in cortical thickness between idiopathic normal-pressure hydrocephalus (INPH) patients and healthy controls. We also explored whether a relationship exists between cortical thinning and gait disturbance in INPH patients. Forty-nine INPH patients and 26 healthy controls were imaged with MRI, including 3-dimensional volumetric images, for automated surface-based cortical thickness analysis across the entire brain. Compared with age- and gender-matched healthy controls, unexpectedly, INPH patients showed statistically significant cortical thickening mainly in areas located in the high convexity of the frontal, parietal, and occipital regions. Additionally, cortical thinning mainly in temporal and orbitofrontal regions was observed in the INPH group relative to the control group. The Gait Status Scale (GSS) scores were negatively correlated with cortical thickness in the medial orbital part of the superior frontal gyrus, gyrus rectus, superior temporal gyrus, temporal pole, and insula. A distinctive pattern of cortical thickness changes was found in INPH patients. We cautiously suggest that cortical thickening in INPH can result from reactive gliosis. Further, our results support the hypothesis that cortical thinning in INPH can result from neuronal degeneration. In addition, cortical thinning can play an important role in gait disturbances in INPH patients.

## Introduction

Idiopathic normal-pressure hydrocephalus (INPH) is a rare disorder in neurology associated with an unknown underlying pathology causing cerebral ventricle enlargement^1^. It is associated with unchanged cerebrospinal fluid (CSF) pressure along with cognitive impairment, gait disturbance, and urinary dysfunction^1^. While patients with INPH can present any combination of the 3 features, the most important clinical feature is disturbance in gait^1^. INPH is underdiagnosed as well as undertreated; possibly only about 20% of INPH patients undergo shunt surgery^2^. Taken together with increased knowledge about INPH, the number of patients in need of shunt surgery for INPH will most likely increase^2^. Many neurosurgical centers recommend the CSF tap test (CSFTT) to diagnose INPH^3^. Further, the CSFTT has a high positive predictive value for successful shunt surgery^3^, and a positive CSFTT response has been regarded as an important predictor of shunt effectiveness and a valuable metric for understanding INPH patients^3^.

When investigating the underlying pathophysiological mechanisms involved in INPH, the cortex is usually overlooked and white matter is often the main focus of consideration^4^. However, some studies suggest that when damage occurs to an axon in the brain, neuronal degeneration not only proceeds distally (Wallerian degeneration) but also proximally (dying back)^5^. Such mechanisms could result in the thinning of cortical areas connected to damaged white matter^5^. A further consideration is that INPH rarely exists in the absence of other neurodegenerative conditions. For example, 89% of INPH cases were also found to have Alzheimer's disease (AD) pathology in a report^6^. Furthermore, AD pathology comorbidity adversely affects shunt surgery outcomes and contributes to INPH symptomatology^7^. And it is well-known that cortical thinning patterns are associated with AD pathology^8^. Therefore, we hypothesized that cerebral cortex degeneration in INPH patients may be as relevant as changes in white matter.

Gait disturbances are common in the elderly, to greater or lesser degrees. In community-dwelling older adults, smaller gray matter volumes in specific regions have been associated with slower gait and poorer balance^9^. And cerebral small vessel disease is mainly characterized by white matter lesions and lacunes^10^. Interestingly, in cerebral small vessel disease, a thinner cortex was also associated with poorer gait^10^. The origin of gait disturbance in INPH remains poorly understood. Furthermore, less is known about the specific role of cortical atrophy in gait disturbances in individuals with INPH. To our knowledge, an investigation of the relationship between cortical atrophy and gait performance in INPH patients has not been reported.

In this study, we investigated differences in cortical thickness utilizing automated surface-based cortical thickness analysis in two groups: INPH patients who had a positive response to the CSFTT and healthy controls. We also explored whether a relationship exists between cortical thinning and gait disturbance in INPH patients. We hypothesized that INPH patients might show a characteristic pattern of cortical thickness change and that there may be unique relationships between cortical thinning and gait disturbance in INPH patients.

## Methods

### Participants

Study participants were recruited prospectively from patients who visited the Center for Neurodegenerative Diseases of Kyungpook National University Chilgok Hospital, South Korea between June 2013 and March 2016. Diagnosis of INPH was made according to the Relkin et al. criteria^1^. Inclusion criteria for patients were as follows: > 40 years of age, 6 months progression or more of gait disturbance plus either cognition or urinary symptoms, and normal CSF opening pressure. Brain MRI revealed ventricle expansion (Evans’ ratio > 0.3) for all patients, with no CSF flow obstruction. Exclusion criteria included patients with a hospitalization history for a significant psychiatric disorder, stroke, a recent history of extensive alcohol use, or a history of metabolic, neurological, or neoplastic dysfunctions that could engender symptoms of dementia. No patient in the study had evidence of intracerebral hemorrhage, head trauma, meningitis, or another potential cause of hydrocephalus.

Criteria for healthy control categorization were as follows: no active neurological, systemic, or psychiatric disorders; normal neurological status in examination; and the ability to function independently. Global cognition of healthy controls was assessed by the Korean-Mini Mental State Examination (K-MMSE)^11^.

This study protocol was approved by the Institutional Review Board of Kyungpook National University Chilgok Hospital. All methods and procedures were performed in accordance with relevant guidelines and regulations. All study participants gave informed and written consent for the study, including information related to clinical data and MRI. Each patient also consented to having a CSFTT.

### Gait assessment and cerebrospinal fluid tap test

Gait disturbance features related to INPH were determined using the Gait Status Scale (GSS)^12^. This scale focuses on 8 factors related to gait disturbance: (1) postural stability, (2) independence in walking, (3) wide base gait, (4) lateral sway, (5) petit-pas gait, (6) festinating gait, (7) gait freezing, and (8) disturbed tandem walking. A total GSS score of the 8 items, ranging from 0 to 16, was determined. A higher score reflected more severe symptoms. The GSS is known to measure overall aspects of walking ability^12^.

A lumbar tap removing 30–50 ml of CSF was performed in all INPH patients. Changes in gait were evaluated repeatedly over 7 days after the tap, while changes in cognition and urination were evaluated at 1 week^13^.

INPH patients having a positive response to the CSFTT were enrolled to increase diagnostic certainty. Response to the CSFTT was defined by 3 scales: INPH grading scale (INPHGS), Timed Up and Go (TUG) test, and K-MMSE. The following criteria were used to identify responders: improvement of 1 point or more on the INPHGS, more than 10% improvement in time on the TUG test, or more than 3 points improvement on the K-MMSE^13^.

### MRI imaging acquisition

MRI data were obtained using a 3.0 T system (GE Discovery MR750, GE Healthcare). Three-dimensional T1-weighted, sagittal, and inversion-recovery fast spoiled gradient echo (IR-FSPGR) MRI images of the whole head, designed to optimally discriminate between brain tissues (sagittal slice thickness 1.0 mm, no gap, TR = 8.2 ms, TE = 3.2 ms, flip angle 12°, matrix size 256 × 256 pixels, and field of view = 240 mm), were acquired^14^.

### Preprocessing and cortical thickness measurement

In order to estimate cortical thickness, the following pipeline image processing steps (CIVET, http://www.bic.mni.mcgill.ca/ServicesSoftware/CIVET) were applied, as described in detail elsewhere^15–21^. MRI in native space was spatially normalized to the stereotaxic space and corrected for intensity non-uniformity artifacts^17^. A multiscale nonlinear registration was then applied to normalize the skull-stripped MR images by a brain extraction tool and to provide a priori information, i.e. tissue probability maps for subsequent tissue classification using a neural network classifier^17^. A trimmed minimum covariance determinant method was applied to estimate and correct partial volume errors which involve MRI intensity mixing at tissue interfaces due to the finite resolution of the imaging device^18^. Cortical surfaces were automatically extracted from each MR volume using Constrained Laplacian-Based Automated Segmentation with Proximities algorithm (CLASP), which reconstructed the inner cortical surface by deforming a spherical mesh onto the white matter (WM)/gray matter (GM) boundary and then expanding the deformable model to the GM/cerebrospinal fluid (CSF) boundary^15^. The extracted surfaces consisted of 40,962 vertices in each hemisphere^15,16^. Finally, the cortical surface models were inversely transformed into the native space so that cortical thickness could be calculated from the Euclidean distance between the corresponding vertices on the WM/GM boundary surface and the GM/CSF intersection surface^16^. And then, an iterative surface registration algorithm was employed to ensure an optimal correspondence at each vertex across individuals^21^. We smoothed them with a Gaussian kernel with a 30-mm full width at half maximum to increase the signal-to-noise ratio and optimally detect population changes^19^.

### Statistical analyses

Statistical analyses were performed with the SurfStat toolbox (http://www.math.mcgill.ca/keith/surfstat) and IBM SPSS Statistics for Windows version 25.0^20^. The demographic data were compared between the INPH and control groups. Fisher’s exact and chi-square tests were used to compare categorical variables, while the Student t tests and Mann–Whitney U tests were used to compare continuous variables. To analyze regional differences in cortical thickness between groups, independent samples t tests were performed on the surface model after matching age and sex in 2 groups. Multiple comparisons were taken into account for the vertex data using a false discovery rate correction at a 0.05 level of significance (FDR, *P* < 0.05). A multiple linear regression analysis was implemented to test localized changes in cortical thickness related to gait disturbance in the INPH group. Cortical thickness was regressed against the GSS score at each vertex using age and sex as covariance parameters according to the equation,

$$ {\text{Y}} = \beta_{0} + \beta_{{1}} {\text{GSS}} + \beta_{{2}} {\text{Age}} + \beta_{{3}} {\text{Gender}} + \varepsilon $$

where Y is the cortical thickness at the vertex, β0 is the Y intercept, β_1-3_ are the regression coefficients, and ε is the residual error.

## Results

A CSFTT was performed on all 64 patients with INPH. Fifteen INPH patients were excluded due to a negative CSFTT response. The final sample for analysis was 49 INPH patients who had a positive response to the CSFTT and 26 age- and gender-matched healthy controls. Table 1 lists clinical and demographic features for INPH patients and control subjects. There were no significant differences in age and gender distribution between the two groups. INPH patients had statistically lower K-MMSE scores than the control subjects.

**Table 1:** Demographic data of INPH patients and controls at baseline (data were collected before CSFTT for INPH patients).

Characteristics	INPH (n = 49)	Control (n = 26)	*P* value
Gender, male	30 (61.2)	10 (38.5)	0.060
Age (year)	73.5 ± 5.4	71.7 ± 4.1	0.065
Education (year)	8.4 ± 5.3	11.1 ± 5.4	0.047
Apolipoprotein E ε4+/ε4−	5/31		
K-MMSE	20.0 ± 5.7	27.2 ± 2.3	< 0.001
GSS	7.6 ± 2.6		

Values denote number (%) or mean ± standard deviation.

*INPH* idiopathic normal-pressure hydrocephalus, *CSFTT* cerebrospinal fluid tap test, *K-MMSE* Korean version of Mini-Mental State Examination, *GSS* Gait Status Scale.

### The comparison of cortical thickness in INPH versus healthy controls

**Figure 1:**
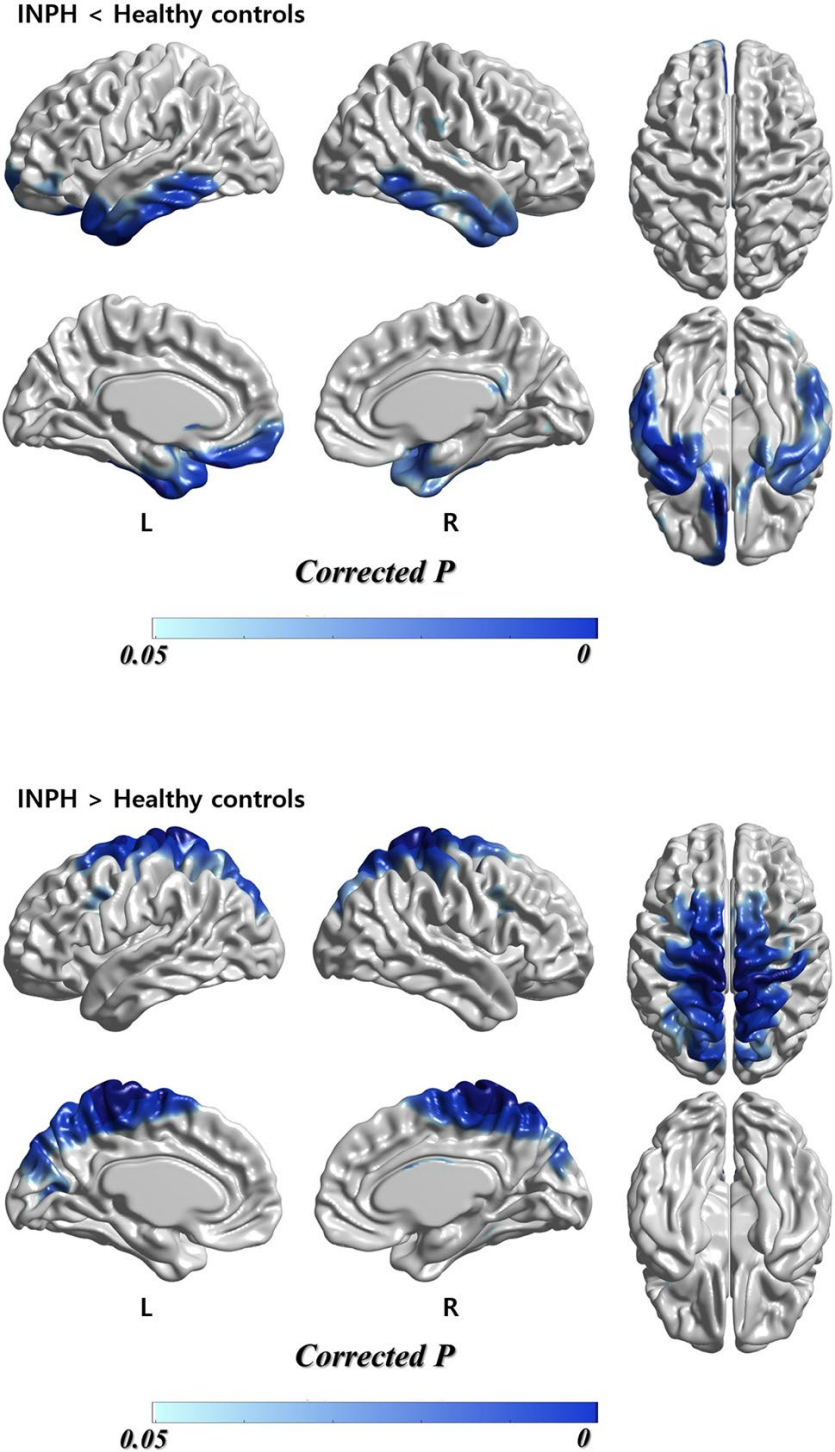
Regions of significant difference in cortical thickness between INPH patients and healthy controls. Statistical significance was set at the level of false discovery rate corrected *P* < 0.05. *INPH* idiopathic normal-pressure hydrocephalus.

A significant localized cortical thinning in INPH patients was seen in the lateral surface of the temporal lobe (right superior temporal gyrus, right Heschl gyrus, bilateral middle temporal gyrus, bilateral inferior temporal gyrus), the orbital surface of the frontal lobe (left orbital part of the superior frontal gyrus, left medial orbital part of the superior frontal gyrus, bilateral gyrus rectus), and the limbic lobe (bilateral temporal pole, bilateral parahippocampal gyrus) (Table 2; Fig. 1). A substantial but localized cortical thinning in INPH patients was also seen in the left fusiform gyrus and right insula (Table 2; Fig. 1).

**Table 2:** Brain regions showing a significant change in cortical thickness using automated surface-based cortical thickness analysis on INPH patients, compared with controls.

AAL label	Side	MNI coordinates (mm)			t value at peak vertex
		x	y	z	
**INPH < controls**					
Superior frontal gyrus, orbital part	L	− 13.3441	29.6287	− 20.4016	5.8802
Superior frontal gyrus, medial orbital	L	− 5.0920	31.7299	− 18.2721	3.8471
Gyrus rectus	R	12.3867	20.4167	− 14.6591	3.3969
	L	− 10.8271	29.4514	− 19.4515	5.8808
Superior temporal gyrus	R	45.6887	− 15.0709	− 1.3781	3.6985
Heschl gyrus	R	42.9363	− 19.3283	2.8886	3.4941
Middle temporal gyrus	R	55.1598	− 47.1271	− 5.0753	3.8506
	L	− 45.0293	1.8944	− 38.7950	4.6285
Inferior temporal gyrus	R	55.2281	− 49.7998	− 6.5307	4.0171
	L	− 41.4122	− 7.3936	− 44.2187	6.7110
Fusiform gyrus	L	− 35.1292	− 8.3738	− 44.0941	6.5411
Temporal pole: superior temporal gyrus	R	29.9650	3.8686	− 22.5338	3.7154
	L	− 31.0891	18.5766	− 39.7111	4.7930
Temporal pole: middle temporal gyrus	R	44.0117	11.9570	− 40.9585	3.4253
	L	− 33.7620	14.1866	− 39.9695	4.8589
Parahippocampal gyrus	R	20.1297	− 2.2402	− 33.4692	3.2492
	L	− 31.1506	− 2.0218	− 33.8223	4.4764
Insula	R	38.0828	4.7012	− 20.4475	3.4577
**INPH > Controls**					
Precentral gyrus	R	16.8910	− 20.1658	75.2878	4.3277
	L	− 15.2051	− 27.7097	66.7879	5.2171
Postcentral gyrus	R	14.4552	− 37.1896	78.6438	6.2001
	L	− 9.1934	− 35.1024	78.7959	6.2651
Superior fontal gyrus, dorsolateral	R	12.2789	− 17.1400	69.0823	3.8645
	L	− 18.1194	− 4.4259	71.5490	4.6521
Middle frontal gyrus	R	33.9247	− 1.3880	54.3737	2.8145
	L	− 22.4661	8.4110	51.0797	3.8027
Supplementary motor area	R	10.0360	− 6.1336	68.4031	3.8893
	L	− 9.9315	− 4.9477	69.6016	4.7635
Paracentral lobule	R	7.3499	− 37.6936	78.7139	6.0228
	L	− 6.2403	− 40.6967	76.9401	6.3948
Superior parietal gyrus	R	14.0987	− 45.8018	76.6706	5.7830
	L	− 14.0987	− 45.8018	76.6706	5.5071
Angular gyrus	L	− 41.1132	− 57.6578	46.1287	3.2656
Precuneus	R	13.4494	− 44.3964	77.3371	5.8750
	L	− 13.4494	− 44.3964	77.3371	5.6610
Superior occipital gyrus	R	10.7603	− 87.4747	39.5211	3.2912
	L	− 12.3968	− 87.5935	39.8908	3.7520
Middle occipital gyrus	L	− 31.6555	− 65.4643	35.3210	2.7503
Cuneus	R	9.2163	− 84.9018	39.9622	3.3291
	L	− 11.8367	− 86.7401	40.6739	3.7759
Calcarine fissure and surrounding cortex	L	− 13.1142	− 70.4829	12.9637	3.3379

All reporting peak vertexes survived at the level of a false discovery rate *P* < 0.05.

*INPH* idiopathic normal-pressure hydrocephalus, *AAL* automated anatomic labeling, *MNI* Montreal Neurological Institute, *L* left, *R* right.

Unexpectedly, a significant localized cortical thickening in INPH patients was found in the medial and lateral surfaces of the central regions (bilateral postcentral gyrus, bilateral precentral gyrus), the parietal lobe (left angular gyrus, bilateral superior parietal gyrus, bilateral precuneus), the frontal lobe (bilateral supplementary motor area, bilateral middle frontal gyrus, dorsolateral part of the bilateral superior frontal gyrus, bilateral paracentral lobule), and the occipital lobe (bilateral superior occipital gyrus, bilateral cuneus, left middle occipital gyrus, left calcarine fissure and surrounding cortex) (Table 2; Fig. 1).

### Correlations of cortical thinning and gait decline

**Figure 2:**
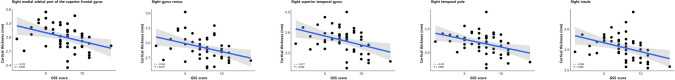
Scatterplots illustrating the relationships between cortical thinning and gait disturbance in INPH patients. *INPH *idiopathic normal-pressure hydrocephalus, *GSS* Gait Status Scale.

Our results in Fig. 2 indicated that GSS scores were negatively correlated with cortical thickness in the right medial orbital part of the superior frontal gyrus (r = − 0.372, *P* = 0.008), right gyrus rectus (r = − 0.365, *P* = 0.010), right superior temporal gyrus (r = − 0.377, *P* = 0.008), right temporal pole (r = − 0.391, *P* = 0.006), and right insula (r = − 0.388, *P* = 0.006).

## Discussion

Compared with age- and gender-matched healthy controls, INPH patients showed statistically significant cortical thickening mainly in areas located in the high convexity of the frontal, parietal, and occipital regions. Additionally, cortical thinning mainly in temporal and orbitofrontal regions was observed in the INPH group relative to the control group. These results provide some evidence for a characteristic pattern of cortical thickness changes in INPH patients.

As an explanation for the cortical thickening in INPH patients, we may speculate as follows. First, cortical thickening has been observed in patients with other neurodegenerative diseases^22,23^. For example, in 96 healthy non-demented volunteers aged 48–75 years, the apolipoprotein E gene ε4 was associated with thicker cortex in several areas^22^. They suggested that the finding of a thicker cortex in ε4 carriers may be an indication of underlying pathological processes, or a response to such processes (e.g., compensation), that increase the risk for developing AD later in life^22^. As another example, a study of AD-associated presenilin-1 mutation carriers showed that asymptomatic mutation carriers also presented increased cortical thickness in the precuneus and parietotemporal areas 9.9 years prior to the predicted age of disease onset^23^. Second, in neurological disorders such as migraine and obstructive sleep apnea, it has been suggested that an increase in cortical thickness could be a consequence of the reactive gliosis that often follows brain pathologies^24–26^. Reactive gliosis is an early inflammatory response characterized by the proliferation of astrocytes and microglia following injury in the central nervous system^27^. Any insult to the central nervous system tissue, including neurodegenerative diseases, also triggers a range of molecular, morphological, and functional changes of astrocytes^28^. And the basic process of reactive gliosis involves cellular hypertrophy, which may be related to the proliferation of astrocytes^29^. Furthermore, reactive gliosis is also known to occur as a compensatory mechanism following damage to the central nervous system, which may be related to cellular hypertrophy^30^. Third, reactive gliosis is well known to be a common phenomenon in hydrocephalus^31^. In hydrocephalus, the stretch and compression of the brain tissue caused by the enlarged ventricles can instigate the proliferation of astrocytes^31^. Interestingly, patients with INPH showed substantial surface expansion primarily in the medial aspects of the frontal horns and the superior portion of the bilateral lateral ventricles, which are surrounded by the high convexity of the frontal and parietal regions and the medial frontal lobe^14^. While INPH patients show substantial ventricular dilatation, CSF space narrowing at the high convexity and high midline areas has been hypothesized to be a characteristic imaging finding in INPH^32^. These areas might be included in the main locations for the disease process in INPH. Furthermore, a previous study about cerebral perfusion patterns in INPH patients highlighted increased perfusion in the frontal, parietal, and occipital areas at high convexity^33^. And brain perfusion and cortical thickness have been generally related^34^. In our study, we cautiously suggest that cortical thickening in INPH may result from reactive gliosis^24,35,36^.

How might the characteristic regional pattern of cortical thinning in our INPH patients be explained? First, in neurodegenerative disease, neuroinflammation often coexists with the degenerative process^37^. Additionally, at a given timepoint, different brain areas can be in different states^38^. Second, cerebral hypoperfusion is known to be related to neuronal degeneration^39^, and is often observed in patients with INPH^33,40^. Regarding cerebral blood flow in INPH patients, many previous studies using single photon emission computed tomography or positron emission tomography have reported a decrease in frontal-dominant perfusion^40^. Ventriculomegaly appears to negatively affect vascular supply to specific brain regions^4^. In addition, temporal lobe perfusion measured by single photon emission computed tomography was also lower in the INPH group relative to the control group^33^. Third, cortical thinning is known to be a biomarker of neurodegenerative changes in the brain and CSF stasis in INPH patients may also promote AD-like patterns of brain atrophy^41^. And it has been hypothesized that CSF stasis in humans and in animals may promote amyloid deposition in older subjects^42^. Further, AD and INPH have been reported to have a common physiological basis in regard to CSF circulatory dysfunction and failure^43^. CSF failure to clear potentially toxic metabolites can lead to amyloid peptide accumulation in the brains of AD or INPH patients^43^. The temporal pole and inferior temporal cortex are typically thought to be prominently affected early in the course of AD on the basis of the burden of pathologic accumulation^44^. In our study, cortical thinning in INPH might result from neuronal degeneration^35^.

In this study, gait decline significantly correlated with cortical thinning in the right medial orbital part of the superior frontal gyrus, right gyrus rectus, right superior temporal gyrus, right temporal pole, and right insula. The role of cortical atrophy in gait disturbances in INPH subjects remains poorly understood. It is generally believed that structural decline (i.e., cortical thinning) in specific regions is correlated with clinical decline^45^. There are several findings in the literature supporting our results. Cognition is known to influence gait and balance in elderly people, and executive functions seem to play a key role in this mechanism^46^. The function of gyrus rectus remains unclear, but it appears to be associated with executive function^47^. Additionally, previous research provided evidence supporting a broader role of the superior frontal gyrus in executive functions^48^. Gait involves the coordination of neural networks and complex sensorimotor behavior, and needs sensory input to determine movement and influence gait^49^. Sufficient coordination of ongoing sensory input is essential for adequate mobilization^49^. The insula is an essential regional hub of integration with significant connections to a large network of brain areas that undergo emotional, sensory, motivational, and cognitive functions; thus, the insula takes in significant sensory stimulation from all types of sources^50^. Further, the superior temporal gyrus associated with somatosensory, vestibular, and visual processing^10^. Further studies simultaneously analyzing cortical thinning, cognitive impairment, and gait decline would be needed to confirm our findings. However, the question remains why significant correlations between cortical thinning and gait disturbance were only found in the right hemisphere in our study. The correlations between these would serve as an intriguing area for future research.

Patients with INPH were chosen in sequential order from our INPH registry. Potential bias was reduced by using objective grading scales before and after CSFTTs instead of subjective accounts by patients or their caregivers. Beyond a small sample size, another limitation for this study was that INPH patients with a negative CSFTT response were not included. The reason was to increase certainty of INPH diagnosis by limiting the analysis to CSFTT positive responders. In addition, INPH patients CSFTT non-responders had greater prevalence of additional cerebral comorbidities^11^. The second limitation was that we did not track additional biomarkers of brain injury, including fluorodeoxyglucose positron emission tomography, or biomarkers that tracked neuroinflammation in our study. Consequently, reactive gliosis and neuronal degeneration could not be determined in our study participants. Further, we did not measure AD-specific biomarkers in our study. And AD pathology could not be confirmed in our INPH patients. However, we also think that there might be justification for analyzing automated surface-based cortical thickness in a large study of patients with INPH. To our knowledge, there is no study analyzing differences in cortical thickness (including thickening) utilizing whole-brain vertex-by-vertex analysis between INPH patients and healthy controls.

In conclusion, a distinctive pattern of cortical thickness changes was found in INPH patients. The INPH patients in our study had significantly thicker cortices than control subjects in areas located in the high convexity of the frontal, parietal, and occipital regions. Significantly thinner cortices were observed in the INPH group relative to the control group in temporal and orbitofrontal regions. Further, this study additionally showed that gait decline correlated with cortical thinning in the medial orbital part of the superior frontal gyrus, gyrus rectus, superior temporal gyrus, temporal pole, and insula. Our findings encourage future studies to elucidate the underlying mechanism of cortical thickness changes in INPH patients.

## Data Availability

The datasets generated and analyzed during the current study are available from the corresponding author upon request.
